# The composite effect reveals that human (but not other primate) faces are special to humans

**DOI:** 10.1371/journal.pone.0286451

**Published:** 2023-05-30

**Authors:** Danielle Sulikowski, Simone Favelle, Elinor McKone, Megan Willis, Darren Burke

**Affiliations:** 1 School of Psychology, Charles Sturt University, Bathurst, Australia; 2 School of Psychology, University of Wollongong, Wollongong, Australia; 3 Research School of Psychology, Australian National University, Canberra, Australia; 4 School of Psychology, Australian Catholic University, Sydney, Australia; 5 School of Psychological Science, University of Newcastle, Ourimbah, Australia; Gabriele d’Annunzio University of Chieti and Pescara: Universita degli Studi Gabriele d’Annunzio Chieti Pescara, ITALY

## Abstract

Recognising faces is widely believed to be achieved using “special” neural and cognitive mechanisms that depend on “holistic” processing, which are not used when recognising other kinds of objects. An important, but largely unaddressed, question is how much like a Human face a stimulus needs to be to engage this “special” mechanism(s). In the current study, we attempted to answer this question in 3 ways. In Experiments 1 and 2 we examined the extent to which the disproportionate inversion effect for human faces extends to the faces of other species (including a range of other primates). Results suggested that the faces of other primates engage the mechanism responsible for the inversion effect approximately as well as that mechanism is engaged by Human faces, but that non-primate faces engage the mechanism less well. And so primate faces, in general, seem to produce a disproportionate inversion effect. In Experiment 3 we examined the extent to which the Composite effect extends to the faces of a range of other primates, and found no compelling evidence of a composite effect for the faces of any other primate. The composite effect was exclusive to Human faces. Because these data differ so dramatically from a previously reported study asking similar questions Taubert (2009), we also (in Experiment 4) ran an exact replication of Taubert’s Experiment 2, which reported on both Inversion and Composite effects in a range of species. We were unable to reproduce the pattern of data reported by Taubert. Overall, the results suggest that the disproportionate inversion effect extends to all of the faces of the non-human primates tested, but that the composite effect is exclusive to Human faces.

## Introduction

Faces are biologically and socially relevant stimuli for Humans. They provide information for identifying expression, gender, identity, and speech which are important cues for social interactions. Because faces are so functionally special, questions that have dominated face research ask how information about faces is processed and whether these visual processing mechanisms are specific to faces (see [1–5]) A perhaps more fundamental question, but one which has been much less investigated, is how much like a Human face a stimulus must be to activate a specialised face processing mechanism? We test this question directly, using the face inversion and composite tasks for a range of natural stimuli that vary in the degree to which they approximate a human face.

Recognising faces is widely believed to be achieved using "special" neural and cognitive mechanisms, that are not engaged when other kinds of objects are recognised [6–8] One way in which face recognition appears to differ from object recognition is that when we are presented with an upright face, information from different regions is integrated in a holistic fashion (for reviews see [1, 4–6]) Objects and inverted faces, on the other hand, are processed in a less configural or holistic manner and rely more on featural processing involving the constituent parts of the face or object [9–11]. While there are various definitions of holistic processing, from whole template representations to some combination of features and the configural (spatial) relationships between them [12], there is general consensus on the paradigms used to measure and manipulate holistic information in face processing [13, 14]. Two classic measures of holistic processing are the *inversion effect* in which recognition or discrimination of human faces is much more affected by turning the images upside down than is recognition of other objects (e.g. [15]); and the *composite effect* in which recognition of (usually) the top half of one person’s face is impaired when it is paired with a different-identity bottom half, if the two haves are vertically aligned, but not if they are laterally offset or misaligned (see Fig 5; e.g., [16]).

While inversion and composite effects are commonly used to test holistic processing, the tasks may reflect different aspects of holistic processing. Rezlescu et al. [13] found that while the face inversion effect correlated moderately with the part-whole effect (another task thought to reflect holistic processing), there was no correlation between the inversion and composite effects, or between the part-whole and composite effects, and only the inversion effect reliably predicted face recognition. Together their results suggest that these tasks tap distinct perceptual mechanisms or possibly subtypes of holistic processing. One way in which inversion and composite effects may differ is as measures of the efficiency of a face specific mechanism (Rezlescu et al., [13]). Face recognition efficiency, the ability to extract relevant information from a face stimulus, has been shown to be significantly reduced by inversion, whereas the efficiency of word and house recognition is not [17] That is, the inversion effect reflects the efficiency with which upright faces are processed. The composite effect, however, appears to be a hallmark of a certain type of information necessary for face recognition. Rossion [5] suggests that holistic processing of the kind measured by the composite effect may be a necessary initial step for processing faces, but that the magnitude of the effect is only weakly related to face recognition, if at all [13, 18–22].

The fact that holistic processing seems to be largely restricted to upright human faces (but with some evidence of integration for bodies as well [23]), especially as indexed by the composite effect [24, 25] raises several important theoretical questions. The question that has most occupied face researchers has been testing the idea that face-like processing might be a consequence of extensive expertise categorising stimuli at a subordinate level, and this issue has generated a great deal of data and debate [2, 6, 25–29]. However, a more fundamental, and often overlooked, question in face research is how similar to a Human face must a stimulus be to elicit the hallmarks of visual face processing? Addressing this question is important as it can point to the parameters the visual system might use to characterise stimuli as faces and fully engage face coding mechanisms. And while this question has been investigated at the low-level end of the spectrum of face and face-like stimuli, for example, face percepts detected in image noise can invoke some aspects of face processing but fail to elicit the N170 hallmark of face coding [30], less research has tested this question using real faces closer to that of Humans.

Evidence of an inversion effect for primate faces is mixed. Wright and Roberts [31] tested both Human and rhesus monkeys and for both groups found an inversion effect for Human faces and not monkey faces (monkey face stimuli consisted of a mix of primates including, among others, chimpanzees, gorillas, orangutans, new and old world monkeys, lemurs, and marmosets). Dufour et al. [32] showed an inversion effect for Human and macaque faces with 750ms exposure in an old/new task but when the exposure was reduced to 50 ms an inversion effect was found only for Human faces. This pattern suggests greater efficiency at processing Human compared to monkey faces. Evidence of a composite effect for primate faces is limited. Taubert [33] reports both inversion and composite effects for Human and primate faces, however, issues with the methodology of that study warrant further investigation. Using the “complete design” composite task, Wang et al. [34] found a composite face effect for Human faces and not monkey faces (species not reported) when participants were asked to match the top halves of faces but found a composite face effect for both Human faces and monkey faces when participants were asked to match the bottom halves of faces.

The most direct way to investigate the extent to which a face-like stimulus engages a face specific processing mechanism is to measure the inversion and composite effects for natural stimuli that vary in the degree to which they approximate a human face. We include inversion and composite effects to test both the efficiency and presence (to a threshold required for face recognition) of holistic processing since it is possible that primate faces may be processed with a similar efficiency to Human faces, but not engage a face specific mechanism. In the current study we used the faces of other species (including a range of other primates), which have similar face features in the same first order configural relationship as human faces. This has the advantage of enabling us to simultaneously examine how similar a face needs to be to a human face physically and phylogenetically, the latter of which might provide hints about the evolution of specialised face processing. A potential limitation of this approach is that we have no *quantitative* measure of *how* similar any given face is to a human face, but this is a notoriously difficult, multidimensional quantification problem, and so we have opted for a similarity manipulation that at least captures biologically meaningful similarity/differences.

## Experiment 1—The inversion effect

While most objects are harder to recognise or match when inverted, faces show a disproportionate inversion effect [15]. Although most holistic/configural and even some featural information is more difficult to extract from inverted faces [1], the disproportionate difficulty with faces is typically attributed to an impaired ability to extract the second order relationships (precise metric distances) between the face parts [9, 35].

**Fig 1:**
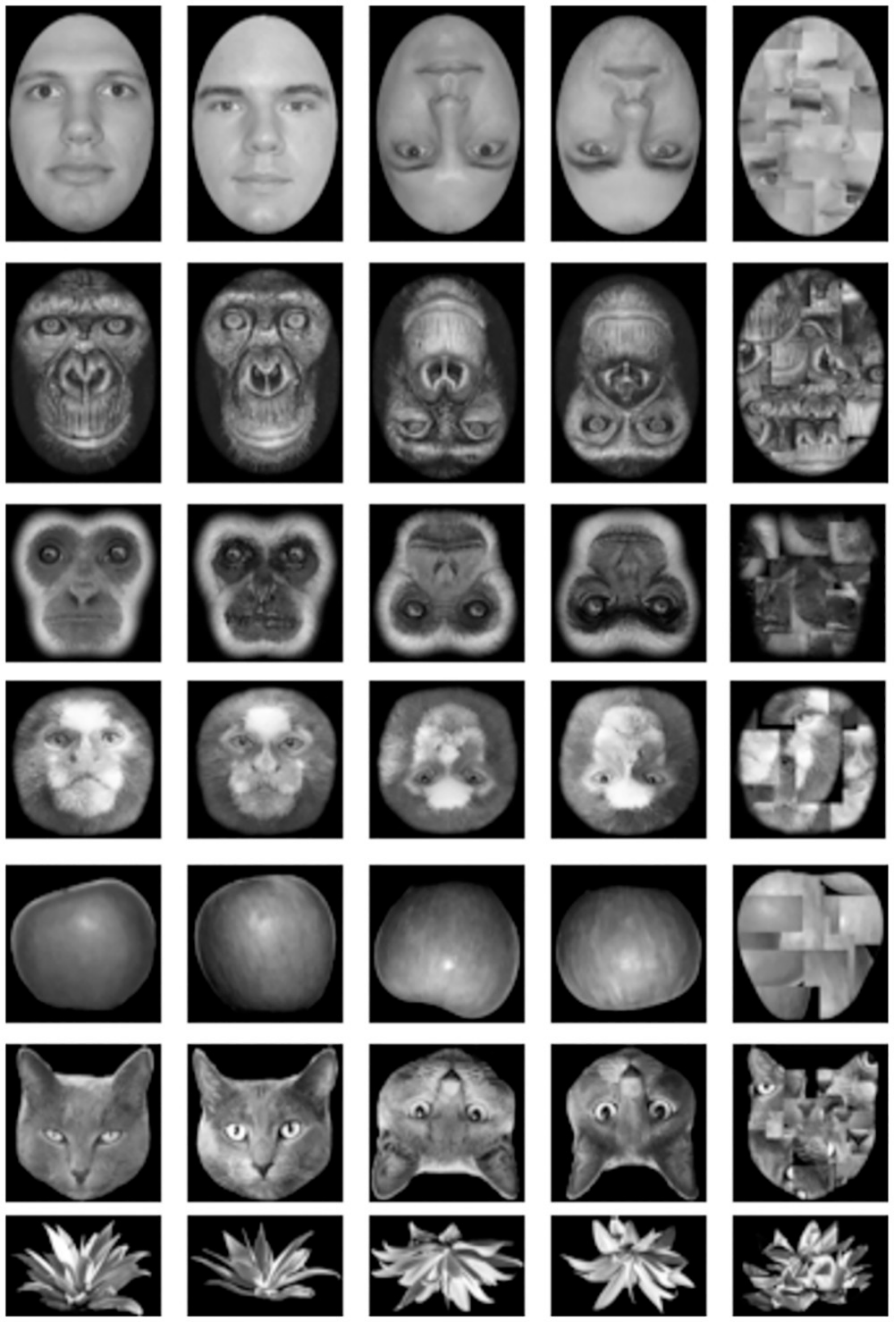
Examples of the stimuli used in Experiment 1.

As a first step in examining how much like a human face a stimulus needs to be to produce a disproportionate inversion effect, we used 5 different kinds of faces (Humans, Common Chimpanzees, Gibbons, Marmosets, and Cats) and two non-face stimuli, selected to not obviously vary on the basis of low-level features (apples and Agave plants). Example stimuli are shown in Fig 1. If the face-specific mechanism can be activated by any kind of face, then all the face stimuli should produce inversion effects equivalent to that produced by human faces, and bigger than that produced by apples and plants. If the quantitative, metric similarity to a human face is important, then the primate faces should produce larger inversion effects than the other stimuli, and chimpanzee faces might produce effects intermediate between that produced by human faces and those produced by the other primate faces.

### Method

#### Participants

Thirty-six undergraduate students (22 female) at the University of Newcastle, whose ages ranged from 18 to 40 (*M* = 20.83, *SD* = 4.91), participated in the experiment for course credit.

Full written consent was obtained from all participants. All procedures were approved by the Human Research Ethics Committee of the University of Newcastle.

#### Stimuli

Four exemplars of each of the 7 stimulus categories were used in the experiment, presented both upright or both inverted (in different conditions), as illustrated in Fig 1. A mask for each stimulus type was also created by randomly selecting segments of each of the 4 exemplars and using them to randomly fill an averaged outline.

The apple and plant stimuli were created for the experiment by taking digital photographs of apples and agave plants and cropping out the backgrounds with Adobe Photoshop. Cat, gibbon, and marmoset faces were sourced online from publicly available Flicker accounts, the human faces were Caucasian male faces from the PAL database (http://agingmind.utdallas.edu/download-stimuli/face-database/)), and the people depicted gave explicit, written permission for their faces to be used in this way, and the chimpanzee faces were scanned from Mollison’s [36] *James & Other Apes*. The stimuli used in the experiment are depicted in Fig 1.

All images were converted to greyscale at a resolution of 28.35 pixels/cm. Within each stimulus category the stimuli were scaled to be of an equivalent size. As there was some variation in the height to width ratio of the various stimulus categories, it was not possible to make all stimuli of an equivalent size. Instead, we ensured that total area of each stimulus was the same across the seven blocks. The human faces were approximately 5cm tall x 3.5cm wide. The stimuli presented on inverted trials had been rotated 180°.

#### Procedure

The sequence commenced with presentation of a fixation cross on a black background for 500 ms. The first stimulus was then presented centrally for 1000 ms. A mask was presented for 500 ms and was both preceded and followed by an inter-stimulus interval of 100 ms. The second stimulus was then presented at one of four randomly determined screen locations and remained on the screen until a response was made. Participants pressed the “S” key to indicate whether the two stimuli were the same or the “K” key if they thought they were different. On half of the trials, the stimuli were presented at an upright orientation, while on the other half the stimuli were presented at an inverted orientation. Each block was composed of 48 randomised trials, half of which were same trials and half were different trials. To make the different trials, each of the 4 exemplars was paired once with each other exemplar upright (12 trials), and once with each other inverted (12 trials). There were also 12 same trials upright, and 12 inverted, with each exemplar paired with itself 3 times upright and 3 times inverted. Trials were presented in random order. Across all blocks, this resulted in a total of 336 trials in the experiment. Prior to completing the experimental trials, participants completed six practice trials using schematic face stimuli.

Stimulus presentation was controlled using SuperLab (Cedrus Corp.) and viewed on an Apple iMac, at a viewing distance of approximately 50 cm. Performance was assessed using a same-different task. Participants were instructed that on each trial they would be shown two exemplars of the stimuli sequentially. They were advised that on each trial the stimuli would either be presented in an upright or inverted orientation. Their task was to decide whether the stimuli were the same or different. Across the experiment, participants completed seven blocks, with each block comprising a different stimulus (i.e., human face, chimpanzee face, gibbon face, marmoset face, cat face, apple, plant). Block order was randomised for each participant.

### Results

**Fig 2:**
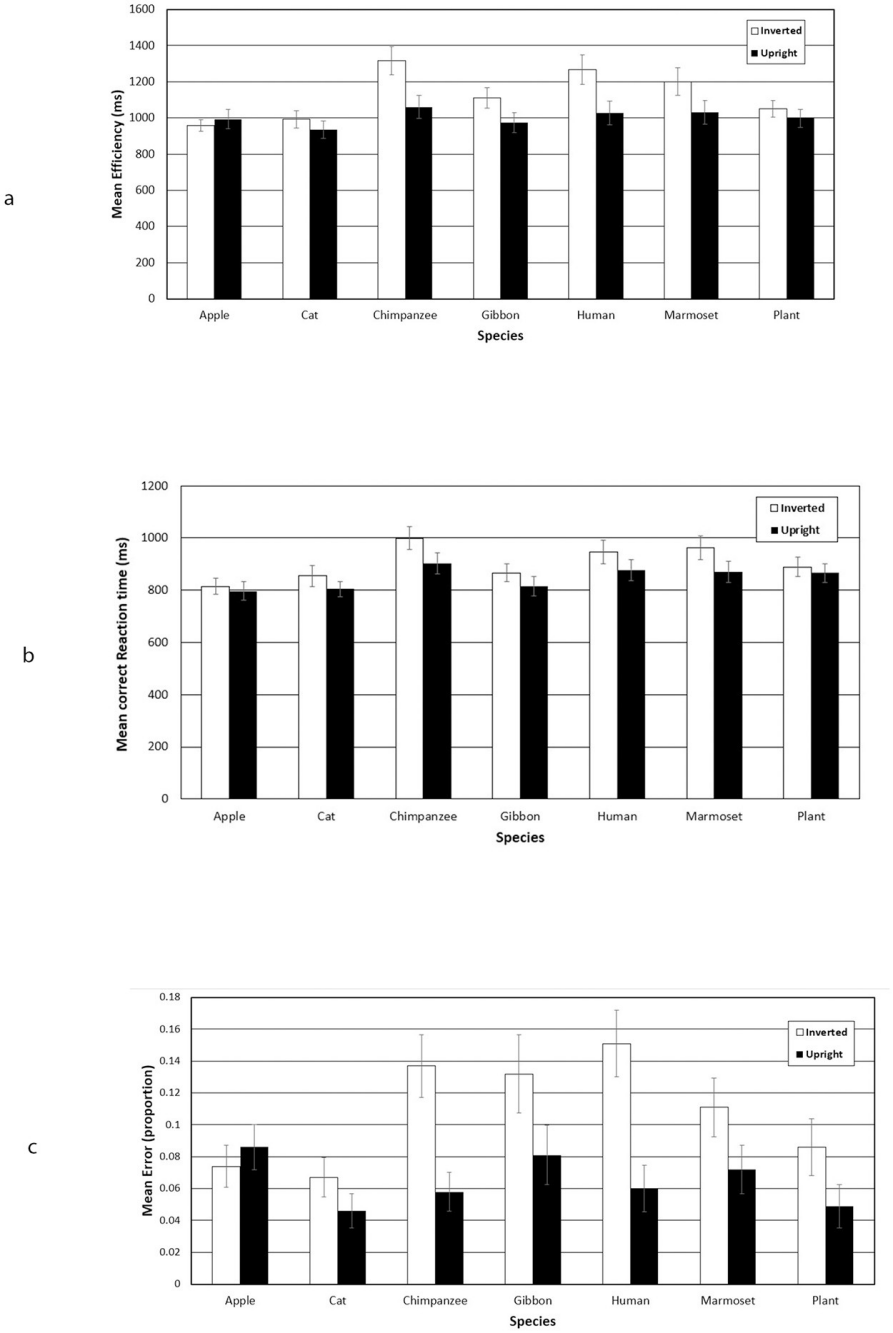
Results from Experiment 1. Error bars represent ±1 SE of the mean.

The primary analysis was a general linear model ANOVA with the within subjects factors of Orientation (inverted, upright) and Species (human, apple, cat, chimpanzee, gibbon, marmoset, plant). This analysis was performed on an efficiency measure and the standard dependent measures of mean percentage error and mean correct RT data (excluding responses two SDs greater than the mean for the condition). We calculated an efficiency measure, in order to account for any possible speed/accuracy tradeoffs. As in previous studies [37, 38]; we calculated inverse efficiency (where a higher value reflects poorer performance) for each condition by dividing mean RT by the proportion of correct trials. The Greenhouse-Geisser epsilon adjusted value is reported in all instances where the sphericity assumption was violated. The results are illustrated in Fig 2.

#### Efficiency

The analysis revealed significant main effects of Orientation, *F*(1, 35) = 52.84, *p* < .001, *η*_*ρ*_^*2*^ = .60, and Species, *F*(4.50, 157.39) = 5.63, *p* < .001, *η*_*ρ*_^*2*^ = .14, which were moderated by a significant Orientation × Species interaction *F*(4.56, 159.66) = 5.84, *p* < .001, *η*_*ρ*_^*2*^ = .14. Significant inversion effects emerged for Humans (t(35) = 3.74, p = 0.001), Chimpanzees (t(35) = 5.27, p < 0.001), Gorillas (t(35) = 3.27, p = 0.002), and Marmosets (t(35) = 3.79, p = 0.001), but not for Cats (t(35) = 1.56, p = 0.129), Apples (t(35) = -0.99, p = 0.329) or Plants (t(35) = 1.49, p = 0.143). In order to examine if the inversion effect was larger for human faces compared to the six other species, we calculated difference scores for each species (inverted—upright) and then performed six planned pairwise comparisons (one-tailed, Bonferroni adjusted), in which we compared the difference score obtained for human faces to that calculated for each of the six other species. These comparisons revealed that a significantly larger inversion effect was observed for human faces compared to cat faces, apples and plants, *t*(35) > 2.52, *p* < 0.05, *d* > 0.57, for all comparisons. In contrast, there was no significant difference between the size of the inversion effect observed for human faces compared to chimpanzee, gibbon and marmoset faces, *t*(35) < 1.64, *p* > 0.333, *d* < 0.33, for all comparisons.

#### Errors

Analysis of errors also revealed significant main effects of Orientation, *F*(1, 35) = 29.21, *p* .001, *η*_*ρ*_^*2*^ = .45, and Species, *F*(4.11, 143.86) = 2.53, *p* = .042, *η*_*ρ*_^*2*^ = .07, along with a significant Orientation × Species interaction *F*(6, 210) = 3.73, *p* = .003, *η*_*ρ*_^*2*^ = .10. Significant inversion effects emerged for Humans (t(35) = 4.33, p < 0.001), Chimpanzees (t(35) = 4.26, p < 0.001), Gorillas (t(35) = 2.26, p = 0.031), Marmosets (t(35) = 2.30, p = 0.027), and Plants (t(35) = 2.09, p = 0.044), but not for Cats (t(35) = 1.39, p = 0.173), or Apples (t(35) = -0.74, p = 0.464). As with efficiency, we performed planned pairwise comparisons between difference scores in order to ascertain if a larger inversion effect was observed for human faces compared to the six other species. Again, a significantly larger inversion effect was observed for human faces compared to cat faces and apples, *t*(35) > 3.00, *p* < .05, *d* > 0.63, for all comparisons. However, there was no significant difference observed between the size of the inversion effect observed for human faces compared to plants, along with chimpanzee, gibbon and marmoset faces, *t*(35) < 2.28, *p* > .087, *d* < 0.46, for all comparisons.

#### RTs

A significant main effect of Orientation emerged, *F*(1, 35) = 51.26, *p* < .001, *η*_*ρ*_^*2*^ = .59, reflecting slower response times on inverted trials (*M* = 905) compared to upright trials (*M* = 848). A significant main effect of Species was also observed, *F*(6, 210) = 7.05, *p* < .001, *η*_*ρ*_^*2*^ = .17. In contrast to our analyses of Efficiency and Errors, the Orientation × Species interaction failed to reach significance, *F*(4.31, 150.77) = 1.66, *p* = .157, *η*_*ρ*_^*2*^ = .05. Significant inversion effects emerged for Humans (t(35) = 2.63, p = 0.013), Chimpanzees (t(35) = 5.47, p < 0.001), and Marmosets (t(35) = 3.19, p = 0.003), but not for Gorillas (t(35) = 2.02, p = 0.051), Cats (t(35) = 1.87, p = 0.070), Apples (t(35) = 0.86, p = 0.395) or Plants (t(35) = 1.49, p = 0.145).

### Discussion

Across the three measures (Efficiency, RT and Errors), there is only reliable evidence of an inversion effect for the primate faces (including Humans), with some suggestion that the effect might be largest for Human, Chimpanzee, and perhaps marmoset faces. Gibbon faces produced large inversion effects in the error measure, but not in the reaction time measure, suggesting some trading of accuracy for speed on the inverted trials. These results suggest that the disproportionate inversion effect for Human faces might extend to the faces of other primates as well, although it might be strongest for the faces of our closest relatives, the chimpanzees. To probe this possibility in more detail, in Experiment 2 we ran a second inversion experiment using a wider range of primate faces.

## Experiment 2—Inversion effect with primate faces

### Participants

Forty one undergraduate students from the University of Newcastle and Charles Sturt University (26 female) participated in the experiment for course credit. Their ages ranged from 18 to 40 (*M* = 21.7, *SD* = 4.28).

Full written consent was obtained from all participants. All procedures were approved by the Human Research Ethics Committees of the University of Newcastle and of Charles Sturt University.

### Stimuli

**Fig 3:**
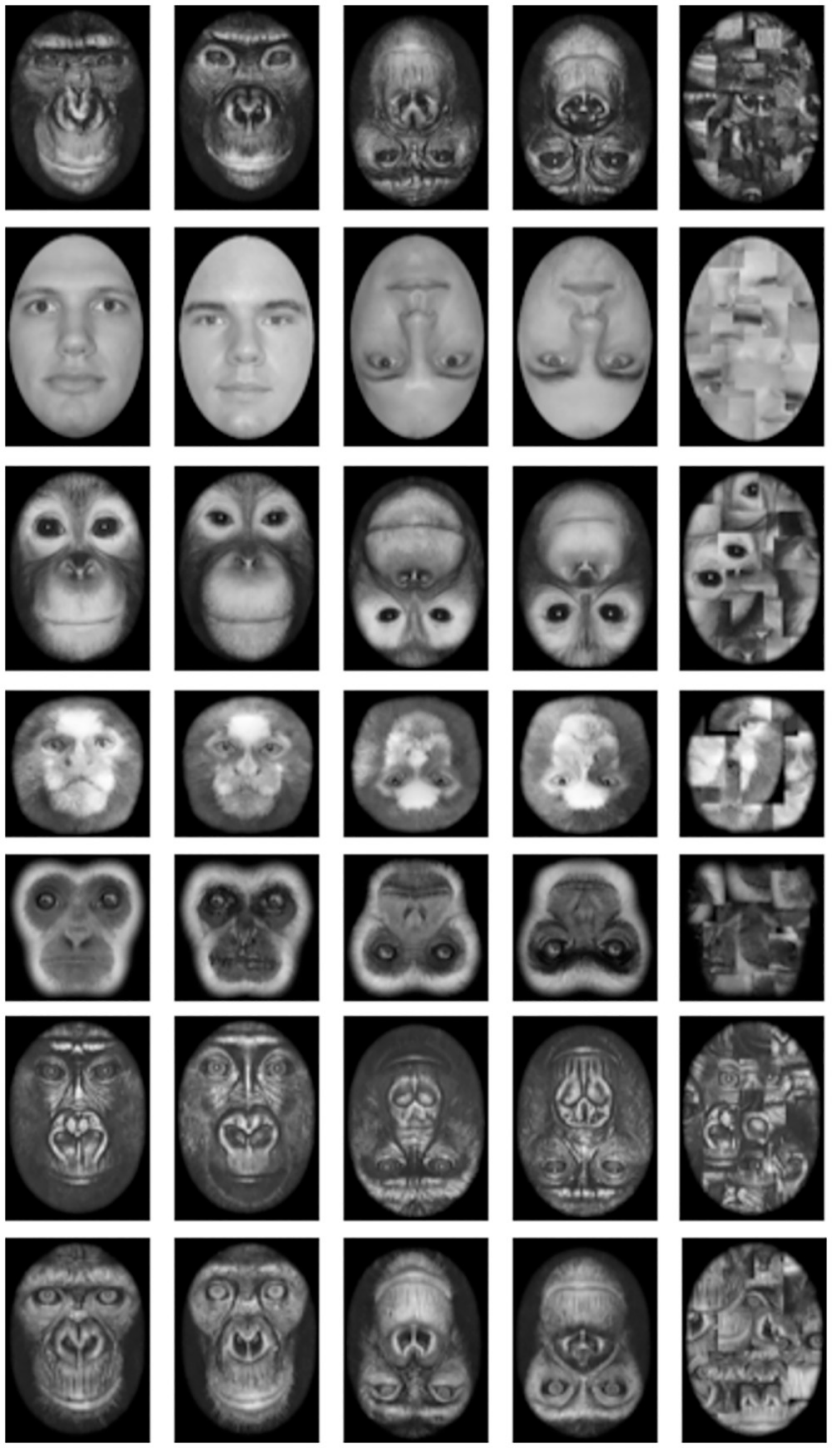
Examples of the stimuli used in Experiment 2.

In addition to the Human, Common Chimpanzee, Marmoset and Gibbon faces used in Experiment 1, in this experiment we also included the faces of Orangutans, Bonobos (Pygmy Chimpanzees), and Lowland Gorillas. The additional primate stimuli were sourced from Mollison (2004), and converted to greyscale in the same way as in Experiment 1. Example stimuli can be seen in Fig 3.

### Procedure

The procedure was largely the same as that described in Experiment 1. The only difference being that in this experiment, participants completed seven blocks, with each block comprising faces of a different primate species (Human, Bonobo, Chimpanzee, Gibbon, Gorilla, Marmoset and Orangutan). Block order was randomised for each participant.

### Results

**Fig 4:**
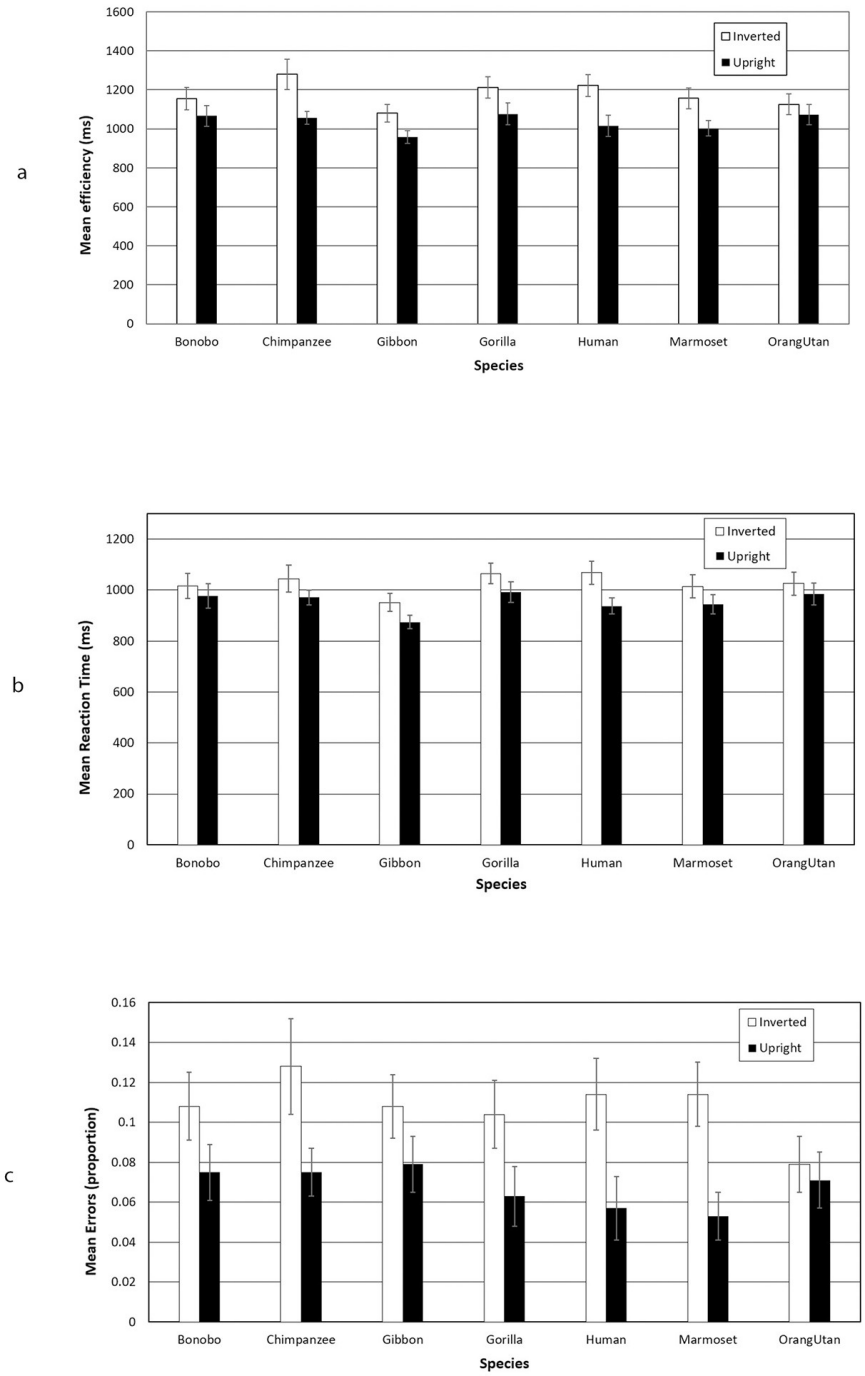
Results from Experiment 2. Error bars represent ±1 SE of the mean.

As in Experiment 1, we analysed mean efficiency, mean proportion error and mean correct RT data (with responses 2 SDs above the mean excluded for each condition). We performed general linear model ANOVAs with the within subjects factors of Orientation (inverted, upright) and Species (human, bonobo, chimpanzee, gibbon, gorilla, marmoset, orangutan). The results are illustrated in Fig 4.

#### Efficiency

A significant main effect of Orientation emerged, *F*(1, 40) = 37.06, *p* < .001, *η*_*ρ*_^*2*^ = .48, reflecting more efficient responses on upright trials than on inverted trials. The main effect of Species, *F*(6, 240) = 2.23, *p* = .041, *η*_*ρ*_^*2*^ = .05 was also significant, but the Orientation × Species interaction, *F*(6, 240) = 1.76, *p* = .107, *η*_*ρ*_^*2*^ = .04, failed to reach significance. Significant inversion effects emerged for Humans (t(40) = 3.10, p = 0.004), Chimpanzees (t(40) = 3.71, p < 0.001), Gorillas (t(40) = 3.10, p = 0.004), Marmosets (t(40) = 3.92, p < 0.001), Bonobos (t(40) = 2.14, p = 0.038), and Gibbons (t(40) = 4.02, p < 0.001), but not for Orangutans (t(40) = 1.22, p = 0.269).

#### Errors

Analysis of errors revealed a significant main effect of Orientation, *F*(1, 40) = 22.73, *p* < .001, *η*_*ρ*_^*2*^ = .36, reflecting poorer performance on inverted trials than on upright trials. The main effect of Species and the Orientation × Species interaction were both non-significant, both *Fs* < 1.01, *p*s > .42.

Significant inversion effects emerged for Humans (t(40) = 2.58, p = 0.014), Chimpanzees (t(40) = 2.69, p = 0.011), Gorillas (t(40) = 2.18, p = 0.035), and Marmosets (t(40) = 3.54, p = 0.001), but not for Bonobos (t(40) = 1.65, p = 0.107), Gibbons (t(40) = 1.69, p = 0.099), or Orangutans (t(40) = 0.42, p = 0.679).

#### RTs

A significant main effect of Orientation emerged, *F*(1, 40) = 27.76, *p* < .001, *η*_*ρ*_^*2*^ = .41, due to faster responses on upright trials than on inverted trials. A significant main effect of Species was also observed, *F*(6, 240) = 2.77, *p* = .013, *η*_*ρ*_^*2*^ = .065. The Orientation × Species interaction again failed to reach significance, *F*(6, 240) = 1.44, *p* = .201, *η*_*ρ*_^*2*^ = 0.04.

Significant inversion effects emerged for Humans (t(40) = 3.85, p < 0.001), Chimpanzees (t(40) = 2.17, p = 0.036), Gorillas (t(40) = 3.26, p = 0.002), Gibbons (t(40) = 3.44, p = 0.001), and Marmosets (t(40) = 2.85, p = 0.007), but not for Bonobos (t(40) = 1.78, p = 0.083), or Orangutans (t(40) = 1.55, p = 0.130).

### Discussion

Consistent with the conclusions of Experiment 1, inversion effects were observed for all primate faces, that did not dramatically differ in size from that produced by Human faces, except for Orangutan faces. Despite the lack of a Species x Orientation interaction, there is some suggestion from the means comparisons that some primate faces may produce smaller inversion effects than others, and there is no compelling evidence of an inversion effect for the Orangutan faces in the current study. There is nothing about Orangutan faces that makes the absence of an inversion effect particularly theoretically interesting. They are phylogenetically intermediate of the species used, and not obviously physically less similar to Human faces than most of the other species used. We excluded male Orangutan faces, since males have large facial flanges, providing potential non-face discrimination cues, so there are also no obvious artefactual cues that might have produced smaller inversion effects for these kinds of faces.

Taken together, Experiments 1 and 2 suggest that the *disproportionate* inversion effect shown for Human faces [15], extends to the faces of other primates (but not to cat faces). There may be some differences produced by the faces of different species of primate, but there is no compelling empirical evidence for such differences, and no theoretically clear pattern to the possible differences, and so based on the data collected with the faces used, the most parsimonious conclusion is that all primate faces produce disproportionate inversion effects, except for the faces of Orangutans in the current study. This suggests that the face perception mechanisms responsible for the inversion effect, is engaged approximately equally well by any primate face. Of course, this does not necessarily imply that humans engage exactly the same mechanisms to perceive the faces of other primates as are used to differentiate human faces, since the inversion effect is only one index of the engagement of specialised face processing mechanisms. There may be a number of distinct perceptual mechanisms that constitute face recognition [13]. Another, perhaps clearer, index of the engagement of such mechanisms is the composite effect, and this is examined in Experiment 3.

## Experiment 3—The composite effect

**Fig 5:**
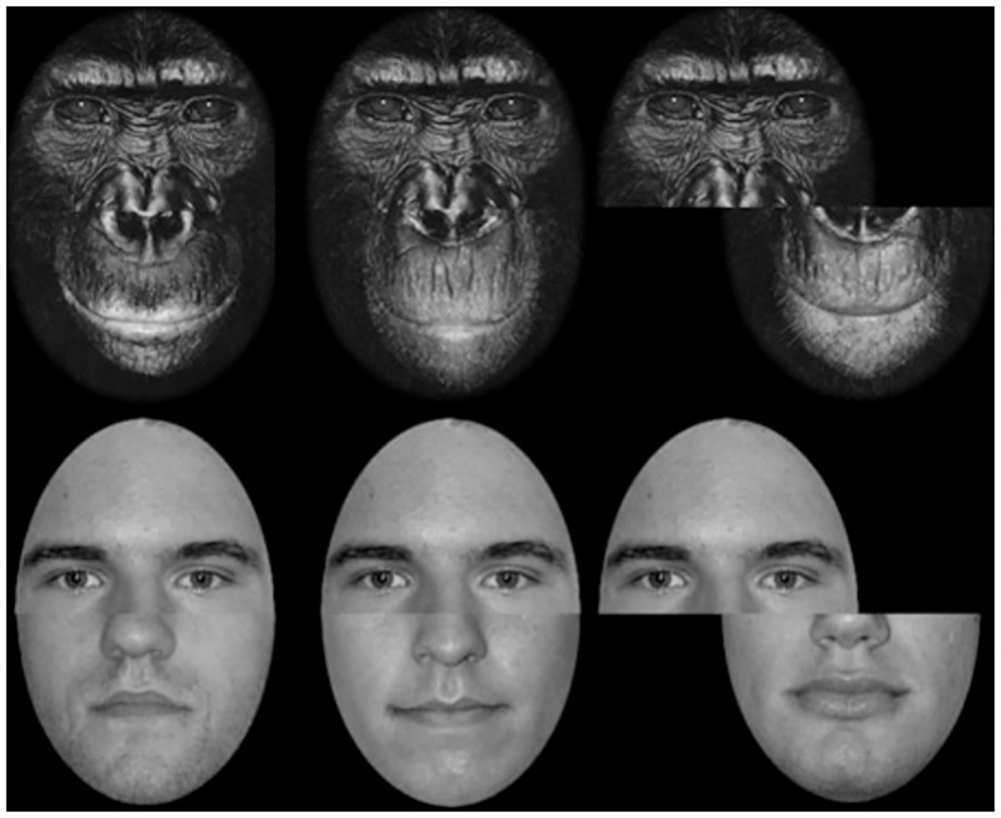
Examples of the human and bonobo composite faces. Depicted are examples of the same top half face aligned (left two images) and misaligned (rightmost images) with different bottom halves.

The composite effect [16] refers to the fact that when one half of a face (typically the top) is presented aligned with the other half (typically the bottom) of a different face, then the top half is much harder to recognise than if the bottom half is presented misaligned (typically offset to one side)–see Fig 5 for examples of the aligned and misaligned face halves used in the current study. This is a reflection of our tendency to process faces holistically, because when the face halves are aligned, they are integrated into a new identity incorporating both halves, and that makes detecting the identity of just the top half of the aligned face difficult. When the face halves are misaligned, then the holistic, integrative mechanism is not engaged (or at least the two halves are not integrated), and so the identity of the top half is much easier to extract. Experiment 3 is designed to examine the extent to which the composite effect extends to the faces of other primates, using the same range of species examined in Experiment 2.

### Methods

#### Participants

Fifty participants (38 female, ages range 18–44, *M* = 21.83, *SD* = 4.88) from the University of Newcastle, and Charles Sturt University took part for partial course credit.

Full written consent was obtained from all participants. All procedures were approved by the Human Research Ethics Committees of the University of Newcastle and of Charles Sturt University.

#### Stimuli

The face stimuli used were the same as those for Experiment 2, except that each face was split just above the nostrils, to create two face halves (a top and a bottom) that could be paired up for the aligned and misaligned trials used to measure the composite effect. For each face type (species), there were 4 individuals, and every combination of non-matching top and bottom halves (12 in total) was used, both aligned and misaligned, making 24 different combinations for each species. Half of the misaligned stimuli had the bottom half offset to the right of the top half (as in Fig 5) and the others were offset to the left.

#### Procedure

Participants were asked to respond as quickly and as accurately as possible in a successive same/different task, deciding whether the top halves of two sequentially presented faces were the same or different. They used the “z” key to indicate a “same” judgement and the “m” key to indicate “different”. Each trial commenced with a 1000 ms white central fixation cross, followed by a 100ms black screen, the first face for 300ms, another 100ms black screen, and the second face for 300ms. A response triggered the commencement of the next trial. The faces to be judged appeared in different randomly selected locations on the screen, and typically required an eye movement to make a judgement. Bottom halves of faces were always different identities from the top halves (potentially producing a new composite identity in aligned trials), and there were 24 unique same (top halves) and 24 different (top halves) trials in the aligned condition, and matching pairs of 24 of each in the misaligned trials, making 96 trials with each species. Practice trials with schematic face halves preceded experimental trials, and judgements were run in species blocks, within which were aligned vs misaligned blocks. Within a species block, aligned blocks always preceded misaligned blocks. The idea of running aligned blocks first was to maximise the composite effect for each species of face, since this may have been important for being able to find differences in the size of the effect for different species of face. Running aligned conditions first means that participants *first* tried to identify the top half of the face in the presence of interference from a different bottom half, rather than in the absence of that interference, as occurs in the misaligned condition. Species block were run in a different randomly generated order for each participant.

### Results

**Fig 6:**
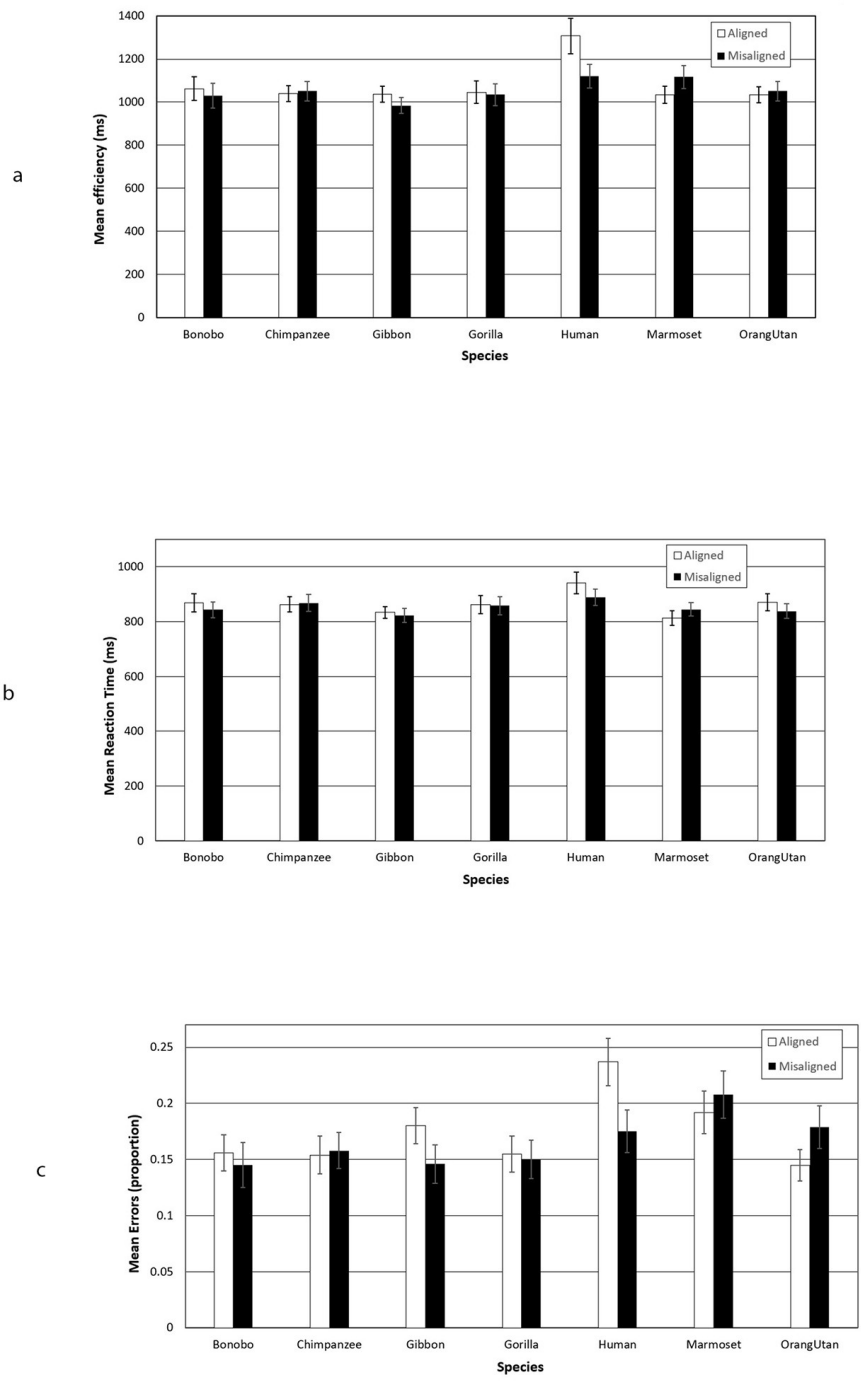
Data from Experiment 3. Error bars represent ±1 SE of the mean.

As in Experiment 2, we analysed mean efficiency, mean proportion error and mean correct RT data (with RTs 3 SDs above the mean excluded). We performed general linear model ANOVAs with the within subjects factors of Alignment (aligned, misaligned) and Species (human, bonobo, chimpanzee, gorilla, orangutan, gibbon, marmoset). Means for all three analyses are plotted in Fig 6.

#### Efficiency

This analysis revealed a significant main effect of Species, *F* (6, 294) = 5.61, *p* < .001, *η*_*ρ*_^*2*^ = .10, and a significant interaction between Species and Alignment, *F* (6, 294) = 4.42, *p* < .001, *η*_*ρ*_^*2*^ = 0.08, but no main effect of Alignment, *F* (6, 294) = 2.82, *p* = .099, *η*_*ρ*_^*2*^ = .05. Pairwise comparisons revealed a significant effect of Alignment for only the Human faces (*p* = .004). The nearest significant difference in the correct direction for the composite effect for any of the other primate faces was for Gibbon faces (*p* = .086).

#### RT

The ANOVA revealed a significant main effect of Species, *F* (6, 294) = 3.17, *p* = .005, *η*_*ρ*_^*2*^ = .061, and a significant interaction between Species and Alignment, *F*(6, 294) = 2.50, *p* = .023, *η*_*ρ*_^*2*^ = 0.05, but no main effect of Alignment, F (1, 294) = 2.80, *p* = .101, *η*_*ρ*_^*2*^ = .05. In order to probe the source of the interaction, we performed pairwise comparisons based on estimated marginal means for each aligned vs misaligned pair (for each Species). The only significant effect of Alignment was for Human faces (*p* = .020). The next closest difference was for Orangutan faces (*p* = .057), all other comparisons had *p* > .105.

#### Errors

The analysis of Error rates also produced a significant main effect of Species, *F* (6, 294) = 5.29, *p* < .001, *η*_*ρ*_^*2*^ = 0.10, and a significant interaction between Species and Alignment, *F* (6, 294) = 4.19, *p* < .001, *η*_*ρ*_^*2*^ = 0.08, but no main effect of Alignment, *F* (6, 294) = 1.28, *p* = .263, *η*_*ρ*_^*2*^ = 0.03. In order to probe the source of the interaction, we again performed pairwise comparisons based on estimated marginal means for each aligned vs misaligned pair (for each Species). In this analysis, significant effects of Alignment emerged for Human faces (*p* = 0.002), Gibbon faces (*p* = 0.036) and Orangutan faces (*p* = 0.026). The significant difference with the Orangutan faces is, however, in the opposite direction to the composite effect (errors are greater on misaligned than aligned trials), suggesting a potential speed/accuracy tradeoff with these faces.

### Discussion

With the stimuli and procedures used here, *only* Human faces produced a reliable composite effect, suggesting that, for Humans, the mechanisms responsible for generating the composite effect is only engaged by Human faces. This contrasts with the findings from Experiment 2, which suggested that all primate faces (even those distantly related to Humans) produced a disproportionate inversion effect. The theoretical implications of this dissociation are important, but will be deferred until the General Discussion. Since our data differ substantially from a previously published report of similar experiments (Taubert, [33]), we first report on an unsuccessful attempt to directly replicate those findings.

## Experiment 4—An unsuccessful exact replication of Taubert (2009)

**Fig 7:**
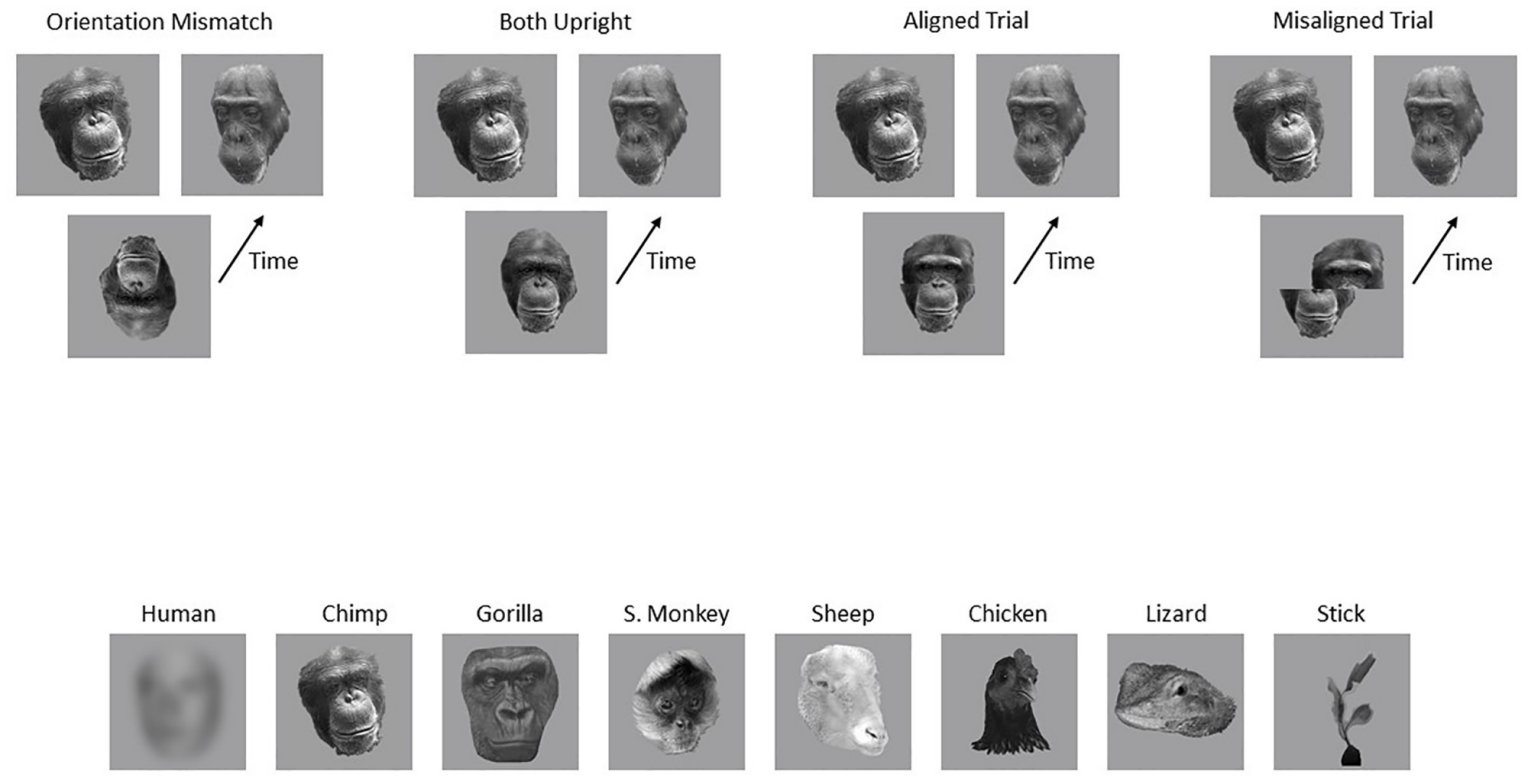
The procedure and example stimuli used in Taubert [33], and in the current replication.

Taubert [33] ran a similar study to those reported here, in which she examined both the inversion effect and the composite effect across a range of different stimuli. She tested Human, Gorilla, Chimpanzee and Monkey faces (spider monkeys—a New World species), as well the faces of hens, Jacky lizards and sheep, and constructed stick objects (see Fig 7 for example stimuli).

Taubert [33] reported strong evidence of both an inversion effect and a composite effect for Human and Chimpanzee faces, but not for any of the other stimuli (although there is a hint of a composite effect for Gorilla faces in the RT data), and concluded that Chimpanzee faces have a “special” processing status for Humans, but that this does not extend to the faces of other primates.

There are a number of aspects of the stimuli and procedures used in the study by Taubert that call into question the pattern of results obtained. The tasks were designed to be directly comparable to data in similar experiments being collected with Spider Monkey subjects, and so were run as a match to sample task, as shown in Fig 7. For both the composite and inversion tasks, participants saw a sample stimulus for 1000ms, which was replaced by two spatially separated choice stimuli, and participants had to press the “v” key if the sample appeared on the left and the “m” key if it appeared on the right. The biggest concern is that in both the inversion task and the composite task, one of the two conditions of interest involve a mismatch of orientation and of alignment, respectively. That is, the inversion task compared performance on a both upright condition with that on an orientation mismatch condition (in which the sample was inverted but the choice stimuli were upright). The composite task used aligned and misaligned sample stimuli but the choice stimuli were always both aligned. That is, the composite task compared performance on a condition in which the sample and both choice stimuli were aligned with a condition in which the sample was misaligned and both choice stimuli were aligned. This design makes the interpretation of the results difficult as any differences in performance between conditions may be due to processes involved in compensating for the orientation or alignment mismatch in one condition and not the other. The other unusual aspect of the study was the stimuli used, which were constructed from photographs taken by Taubert. In both the inversion and composite tasks, the matching stimulus (the whole face for inversion, or the top half for composite) was a different image (frequently slightly rotated) of the same individual, rather than being the same image, as is typical. There was also no attempt to control the sex of the face being judged. The human stimuli contained images of 2 different males and 3 different females.

Rather than try to determine how the unusual aspects of Taubert’s [33] procedure and stimuli may have contributed to the differences between our results and hers, we instead ran a direct replication of her Experiment 2 with new groups of participants.

### Methods

#### Participants

Twenty-one participants from Wollongong University, the University of Newcastle and Charles Sturt University (15 female) participated in the replication of the Inversion experiment and 58 participants from the same populations (19 male) participated in the replication of the Composite experiment.

Full written consent was obtained from all participants. All procedures were approved by the Human Research Ethics Committees of the University of Wollongong, the University of Newcastle, and of Charles Sturt University.

#### Stimuli and procedure

These were identical to those used in Experiment 2 by Taubert [33], and illustrated in Fig 7. In order to make the replication as direct as possible, we ran the same SuperLab scripts that she had used to collect the original data, using the apparatus and viewing distances described in our Experiment 1. We were in possession of the SuperLab scripts because the data from Taubert [33] were collected under the supervision of some of the current authors [39], but the other authors subsequently lost confidence in the data.

### Results

**Fig 8:**
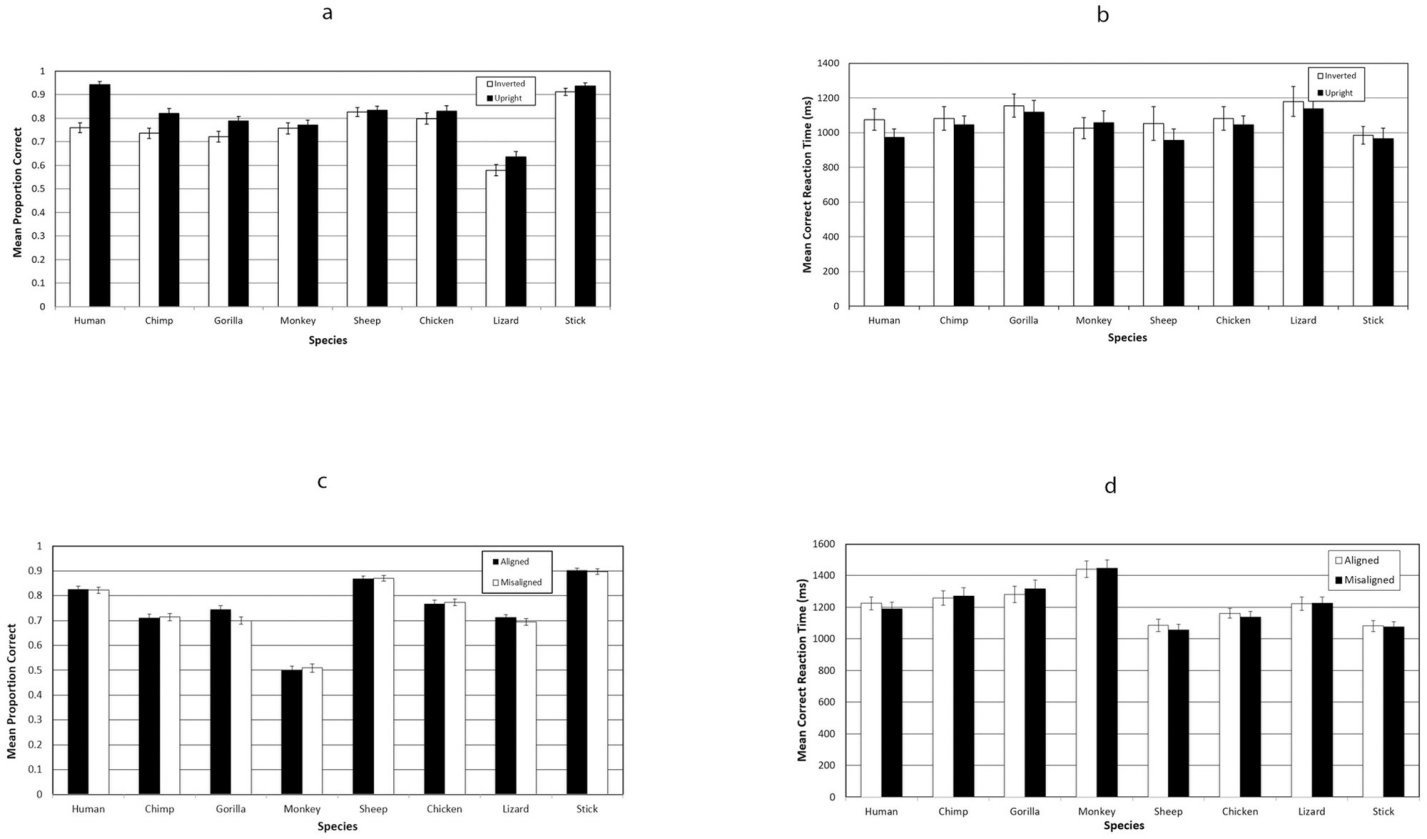
Data from Experiment 4, the direct replication of Taubert [33]. Error bars represent ±1 SE of the mean.

Taubert [33] analysed correct RT and Proportion Correct, and so we analysed the same variables to aid direct comparison with her data. The data are plotted in Fig 8.

#### Inversion effect—RT

The data were analysed using a General Linear Model Repeated Measures Factorial ANOVA with within subjects factors of Species (8 levels) and Inversion (upright, inverted). This analysis revealed a significant main effect of Species, *F*(7,140) = 7.12, *p* < .001, a significant main effect of Inversion, *F* (1, 20) = 4.58, *p* = .045, but no significant interaction, *F* (7, 140) = 0.70, and so, in the RT data, there is no evidence to suggest that any species are producing a disproportionate inversion effect. This is not the pattern of results reported by Taubert [33], in which inversion effects were reported for only Human and Chimpanzee faces.

#### Inversion effect—Proportion correct

The same analysis was used for the Proportion Correct data. This analysis revealed a significant main effect of Species, *F*(7,140) = 39.15, *p* < .001, a significant main effect of Inversion, *F* (1, 20) = 57.67, *p* < .001, and significant interaction, *F* (7, 140) = 5.74, *p* < 0.001. The interaction was probed further using pairwise comparisons, and this revealed significant Inversion effects for Human faces (*p* < .001), Chimpanzee faces (*p* = .03), Gorilla faces (*p* = .03), and Jacky lizard faces (*p* = .038). This suggests, that, as in our Experiments 1 and 2, the differential inversion effect extends to the faces of other primates (although not to the monkey faces, here), and shows some evidence, in this data set, of extending (perhaps less strongly) to Lizard faces. This is not the pattern of results reported by Taubert [33], in which inversion effects were reported for only Human and Chimpanzee faces.

#### Composite effect—RT

The data were analysed using a General Linear Model Repeated Measures Factorial ANOVA with within subjects factors of Species (8 levels) and Alignment (aligned, misaligned). This analysis revealed a significant main effect of Species, *F*(7, 399) = 48.40, *p* < .001, but no significant main effect of Alignment, *F* (1, 57) = 0.23, and no significant interaction, F (7, 399) = 1.08. There is no evidence from these data that a composite effect occurred for any stimuli.

#### Composite effect—Proportion correct

The same analysis was used for the Proportion Correct data. This analysis revealed a significant main effect of Species, *F*(7, 399) = 152.73, *p* < .001, but no significant main effect of Alignment, F (1, 57) = 1.84, and no significant interaction, F (7, 399) = 1.40. There is no evidence from these data that a composite effect occurred for any stimuli.

### Discussion

Our direct replication of Experiment 2 from Taubert [33] has produced data that in no way match those reported in that study. There is no evidence from the replication that Chimpanzee faces have some kind of special status for Humans, as concluded by Taubert.

In our replication of the Inversion experiment, the data show a pattern that is similar to the Inversion experiments we report here (Experiments 1 & 2), in that Human-sized inversion effects extend to the faces of other primates, with somewhat smaller effects for some other kinds of faces (sometimes Cats in our Exp 1 and Lizards in the current replication), as would be expected if primate faces produced a disproportionate inversion effect. The fact that the inversion effect did not extend to the monkey faces used by Taubert might be a reflection of the quality of the stimuli, and/or of the fact that for these stimuli particularly, participants had trouble generalising between the different images of the same individual. Consistent with either of those possibilities is the fact that in the composite task (which used the same stimuli—albeit split above the nostrils), performance with the monkey faces was at chance.

The replication of the Composite effect from Taubert’s Experiment 2 produced only an effect of species. Participants were faster and performed better on the task for some kinds of stimuli, especially sheep faces and sticks, but there is *no* evidence of a composite effect for any of the stimuli used. This is almost certainly a consequence of the unusual nature of the stimuli and procedure used to attempt to measure the composite effect. As mentioned previously, the match to sample design with aligned or misaligned samples being matched to two aligned choice stimuli means that there is always holistic interference in each trial type. Because we were concerned that this might make any effects difficult to uncover, we ran nearly twice as many participants in our study as Taubert had run, but nevertheless were unable to detect a composite effect for any of the stimuli. The stimuli and procedures used by Taubert [33] show no evidence, here, of producing a measurable composite effect.

## General discussion

Across the 4 experiments reported here we have shown that the faces of other primates engage some forms of holistic processing, but not others. Experiments 1 and 2, as well as the replication of the inversion experiment from Taubert [33] reported in our Experiment 4, all suggest that the disproportionate inversion effect produced when judging Human faces, extends approximately equally well to the faces of all of the other primates we tested, but that it does not extend as well to the faces of non-primates. And so, using the Inversion effect as an index of the efficiency of holistic processing suggests that the mechanism is engaged equally well by all primate faces. Whether this is a consequence of an evolutionarily old face-specific mechanism that is shared by all of the primates, or is a consequence of the faces of other primates simply being similar enough to Human faces to engage the mechanism, is a question that will need to be answered in future research. One obvious way to address that question is to examine the faces to which the Inversion effect generalises for non-human primate subjects. Burke & Sulikowski [40] reviewed the evidence for such effects in non-human primates and concluded that although there is good evidence of disproportionate inversion effects (for own-species and human faces) from both New- [41, 42] and Old-World [43] monkeys, too few other species faces were tested to be sure how primate-face-general the effect might be.

The most interesting aspect of the current study is that, although the disproportionate inversion effect extends to the faces of other primates, the composite effect appears to be genuinely Human-face-specific. This result is consistent with Wang et al. [34] who also found a composite effect for Human and not monkey faces when matching the top halves of faces. Wang et al. [34] also found a composite effect for both Human and monkey faces when matching the bottom halves of faces, but this finding may reflect the involvement of information other than holistic information. The composite effect is not often tested or found using the bottom half of faces and may be confounded by different kinds of information like optimal fixation points and regional salience [5]. That the composite effect is Human-face specific holds a number of important theoretical implications. We do not know whether this effect is particular to humans, because, as reviewed by Burke & Sulikowski [40], there is only one reliable demonstration of a composite effect in a non-human primate [44], using Rhesus Macaque subjects, and they did not test for whether the effect generalised to the faces of other primates. The only experiment that has attempted to measure whether a composite effect in a non-human primate extends to the faces of other species was conducted by Taubert [45], using Spider Monkeys, but the results of that study are not particularly convincing, since performance on aligned trials with Spider monkey faces, for each of the Monkeys tested, was 25%, exactly as far from chance (in a two-alternative match to sample task) as was performance (for both monkeys) on misaligned trials (75%).

The finding that the composite effect is specific to human faces, but the disproportionate inversion effect is not, is consistent with recent discussions questioning the idea that holistic processing in faces is a singular, generic perceptual mechanism [12, 13]. While the inversion task and the composite task are widely used as measures of holistic processing in face research, Rezlescu et al. [13] have shown that they likely reflect distinct mechanisms. Specifically, they argue that the inversion effect measures the efficiency of face processing, but that the composite effect is a hallmark of a kind of information required for processing upright human faces. Our finding of an inversion effect for all the primate faces we tested that was disproportionate to the objects tested suggests similar efficiency of processing primate faces that is not necessarily qualitatively different to processing non-face objects (Piepers & Robbins, 2012). That we found a composite effect only for human faces and not for any other primate face or object supports the idea that holistic processing may be required to be present at some threshold for processing human faces [5, 13].

We have used inversion and composite effects which differ in how well they measure the efficiency of face recognition or the presence of holistic processing. It should be noted that these tasks differ in other ways that may influence performance. The inversion effect involves comparison of performance for normal, whole faces that differ only in orientation. The effect is driven by fewer errors in the upright, whole face condition. The composite effect, on the other hand, is based on an illusion in which identical face halves are perceived as being different. It is a measure of how well holistic processing can be inhibited and relies on greater errors in the whole face (aligned) condition compared to a misaligned or non-face condition. Another factor which may influence performance is that within-category discrimination is typically engaged to a greater extent by Human faces compared to the other stimuli we test. Most people have more experience with seeing and making individuation decisions for Human faces compared to other primate faces. However, while experience may modulate the size of the composite effect in Human faces, for example, larger composite effects for own-age faces [46–48], composite effects were found regardless of experience. That is, a lack of experience does not abolish the composite effect so we would not expect that the lack of a composite effect for other primate faces would be accounted for by a lack of experience.

In the absence of reliable data about the extent to which composite effects in non-humans extend to the faces of other species, we cannot draw any strong conclusions about the uniqueness or otherwise of the human-face-specificity of the Composite effect for human participants. The mere fact that the composite effect, but not the disproportionate inversion effect, occurs only for Human faces, however, suggests that the two effects are tapping different aspects of holistic processing, and that only the Composite effect depends on a mechanism that is genuinely Human-face-specific. The current data do not speak to whether that specificity depends on experience—that is a question for future research—but it does suggest that Human faces are, in at least some respects, genuinely *special* to Human perceivers.
